# The efficacy of gluteal tendinopathy treatments: A systematic review

**DOI:** 10.1177/02692155251327298

**Published:** 2025-04-13

**Authors:** Tobias Bremer, Peter Nicklen, Angie Fearon, Dylan Morrissey

**Affiliations:** 1 Sports and Exercise Medicine, William Harvey Research Institute, 4952 Queen Mary University of London, London, UK; 2 22457 Faculty of Medicine, Nursing and Health Sciences, School of Primary and Allied Health Care, Monash University, Frankston, Australia; 3 Faculty of Health, 2234 University of Canberra, Office 13, Clinical Education and Research Centre, UC Hospital, Canberra, Australia

**Keywords:** Gluteal tendinopathy, Greater trochanteric pain syndrome, GTPS, Lateral hip pain, management

## Abstract

**Objective:**

To guide clinical practice by synthesising robust evidence concerning gluteal tendinopathy management.

**Data sources:**

Five electronic databases were searched from inception to August 2024 for randomised controlled trials (RCTs) of medium or high quality, and low risk of bias, that measured pain and function in adults with clinically diagnosed gluteal tendinopathy.

**Review methods:**

Systematic review reporting proof of efficacy. PEDro scale and Cochrane Risk of Bias Tool 2.0 were used to assess internal validity and risk of bias. Efficacy was determined by comparison to minimal intervention. Methodological heterogeneity prevented meta-analysis, but we calculated standardised mean differences (SMD) and 95% confidence intervals (95% CI) for individual study arms to facilitate comparison between interventions.

**Results:**

Four interventions from four RCTs demonstrated efficacy. Exercise and education has moderate strength evidence of a medium effect on pain (SMD = 0.95; 95% CI [0.58, 1.33]) and function (SMD = 0.91; 95% CI [0.53, 1.28]) in the short term with small effects in the medium and long term. Corticosteroid injection has moderate strength evidence of a small effect on pain (SMD = 0.51; 95% CI [0.16, 0.86]) in the short term. Platelet-rich plasma injection was superior in the short term compared to corticosteroid injection for function (SMD = 0.46; 95% CI [0.00, 0.91]). For pain, focused shockwave therapy (f-ESWT) demonstrates superiority in the long term (SMD = 5.77; 95% CI [4.84, 6.71]) compared to corticosteroid injection.

**Conclusions:**

Exercise and education can be cautiously recommended as the core approach for pain management and function, potentially supplemented by corticosteroid or f-ESWT, while definitive trials of promising interventions are needed to derive robust practice recommendations.

## Introduction

Gluteal tendinopathy is one of the most common reasons for lateral hip pain that presents in general practice.^1^ Patients with severe gluteal tendinopathy report low quality of life, equivalent to people with end-stage hip osteoarthritis as well as similar levels of pain and dysfunction.^2^ Gluteal tendinopathy negatively impacts function to such an extent that those affected are less likely to be in full time employment compared to pain-free individuals and those with hip osteoarthritis.^3^ People affected by gluteal tendinopathy have a higher BMI, lower pain thresholds, strength reduction in hip abduction^4,5^ and higher levels of anxiety and depression when compared to a healthy control group.^6^ The impact that gluteal tendinopathy has on quality of life has also been demonstrated by analysis of the wide variety of biopsychosocial domains affected.^6^

Tendinopathy of the gluteus medius and minimus tendons is likely to be the primary cause of gluteal tendinopathy.^7–10^ It has been suggested that affected structures around the greater trochanter, such as the trochanteric bursa^11^ and iliotibial band,^11^ can be involved secondarily as part of gluteal tendinopathy. Greater trochanteric pain syndrome is used interchangeably with gluteal tendinopathy as a diagnostic label, reflecting the complexity of the localised pathology, heterogeneous nature of clinical presentations and lack of pathophysiological clarity.^12^ Gluteal tendinopathy commonly affects people between the fourth and sixth decades of their life^2,13^ and is more prevalent in females, with the distribution estimated to be 73% female to 27% male.^2^ Gluteal tendinopathy is often characterised by inability to sleep on the affected side, increase in symptoms with usage of stairs, and inability to participate in sports and other recreational activities.^14,15^

Previous systematic reviews^16–20^ have not established the efficacy of interventions compared with sham or placebo or wait-and-see, therefore it is unclear if these interventions have proven efficacy. Firstly, this systematic review establishes the efficacy of interventions compared with sham or placebo or wait-and-see. Secondly, this systematic review compares interventions with proven efficacy against interventions which have shown promise in the literature to establish secondary proof of efficacy, to provide treatment options with proven efficacy for clinicians. Several recent studies have investigated the effects of a variety of interventions for gluteal tendinopathy such as corticosteroid injections and platelet-rich plasma injections,^21,22^ and exercise and education,^15,23^ with positive findings. Further, more invasive interventions such as surgery^24–27^ report good outcomes for both partial and full thickness tears of the abductor complex. However, it remains unclear which interventions have the best efficacy at given time points in a population with heterogeneous aetiologies and variable activity, pain level, and functional status presentations.

This systematic review included any intervention used by any professional tasked with the management of this demographic, thereby adhering to the Appraisal of guidelines for research and evaluation II standards for the development of clinical guidelines.^28^ An evidence map was used to report the effect and outcomes of modalities informing both clinical decision-making as well as further research. When considered alongside clinical reasoning and patient preferences, this systematic review should enable clinicians to design and deliver optimal management of people with gluteal tendinopathy that is in line with best evidence, and avoid conclusions based on poor quality, or biased, studies that have not demonstrated robust proof of efficacy.

## Methods

The systematic review was registered at Prospero (CRD42019140231) on 16 December 2019 and guided by the PRISMA statement for design and reporting.^29^

## Search methods

### Selection criteria for studies

Type of studies – published and non-published, high-quality, randomised controlled trials were eligible for inclusion in our review.

Type of participants – A randomised controlled trial was included if the participants were clinically diagnosed as having gluteal tendinopathy with diagnostic criteria including the presence of pain around the greater femoral trochanter, positive pain provocation testing for gluteal tendinopathy, with/out radiographic confirmation (ultrasound or magnetic resonance imaging). Other documented causes of lateral hip pain were excluded such as intra-articular disorders including osteoarthritis, femoroacetabular impingement or labral lesions; or referred pain from the lower back.

Type of interventions – Any randomised controlled trial that focused on the effectiveness of an intervention in the management of gluteal tendinopathy was included. There was no restriction on the intervention used or language of report, with both surgical and non-surgical interventions being considered.

Type of outcomes – Studies that included at least one of the following outcomes: patient reported functional outcome measures, pain, and global perceived effect scales, were included in the review.

### Search strategy

We searched The National Library of Medicine Washington DC (MEDLINE via Pubmed), the Cochrane Central Register of Controlled Trials (CENTRAL), Exerpta Medical Database by Elsevier (EMBASE), Cumulative Index to Nursing and Allied Health Literature (CINAHL), Web of Science, BIOSIS (OVID) and SPORTDiscus from inception to August 2024. In addition, we searched Controlled-trials.com, ClinicalTrials.gov, and the International Clinical Trials Registry Platform of the WHO for relevant reports that did not reach full publication. The reference lists of all included studies and citing articles were used to identify trials not already identified by our search strategy. Search terms can be found in Appendix 1, MeSH terms were included. Terms for Pathology, Intervention/treatment modality, and Types of trials were grouped with ‘OR’, then combined with ‘AND’.

Title and abstracts found during the search of the electronic databases were uploaded to EndNote reference manager^30^ and duplicates removed. The abstracts were screened for eligibility by two reviewers (PN and TB), with a third reviewer on hand in case of disagreement (DM). Full texts were retrieved and likewise assessed for eligibility, with those suitable for the review proceeding to methodological quality and risk of bias assessment. This was managed in Microsoft Excel.^31^

## Methodological quality and risk of bias assessment

Two reviewers independently assessed the selected studies for methodological quality using the validated PEDro tool.^32^ This tool is made up of eleven categories which were scored dichotomously (yes or no). The first of these was not used to calculate the overall score, as such the highest achievable score is ten with higher scores indicating higher methodological quality. Disagreements between the reviewers were resolved by discussion. If disagreement was persistent, a third reviewer (DM) mediated the decision. Studies that scored less than four out of ten were considered to have poor methodological quality and excluded from the review.

Two reviewers independently assessed the selected studies using the Cochrane Risk of Bias Tool 2.0.^33^ The five domains assessed were randomisation process, deviations from intended interventions, missing outcome data, measurement of the outcome, and selection of the reported result. Each domain was assessed as low (+), some concern (?), or high (−) risk of bias, based on the Cochrane criteria^34^ and an overall score assigned. Where an item could not be scored due to lack of reporting that criteria was scored as having ‘no information’. Studies that were at high risk of bias were excluded from contributing to the recommendations.

## Data extraction and analysis

### Data extraction

Pairs of investigators (PN, TB, AF, and DM) extracted data independently to reduce the risk of error. Two reviewers (PN and TB) then resolved any discrepancies and collated the extracted data for reporting. The data for participant characteristics, interventions (type, duration, number of sessions), outcome measures, and outcome data were extracted.

### Determining efficacy and data synthesis

The efficacy and strength of evidence was evaluated and presented in an ‘evidence map’ (Table 4). Interventions were considered to demonstrate efficacy if included trials demonstrated (i) primary proof of superiority compared with sham or placebo or wait-and-see or (ii) secondary proof of superiority compared with another treatment of proven efficacy.^35^ One study^36^ allowed for between arm comparison of two interventions, corticosteroid and education and exercise, to a wait and see group to establish primary proof of efficacy. Secondary proof of efficacy was established by comparison of individual interventions which had shown promise in the literature, for example F-ESWT^37^ and PRP^22^ and had proven methodological quality and were not at high risk of bias.

The results are reported as (SMD) with confidence intervals calculated in RevMan 5,^38^ using a random effects model for caution, to standardise the results description and enable comparison between studies. Ninety-five percent confidence intervals were presented to demonstrate the certainty of the treatment effects.

Pooling and meta-analysis to determine relative effects of individual treatments or the outcomes for a given intervention across multiple studies was not possible due to methodological heterogeneity (see Appendix 2).

The strength of the evidence^39^ and effect size^40^ was separately determined for pain and function outcomes for each intervention. Strong evidence was defined by consistent findings in multiple high quality randomised controlled trials. Moderate strength evidence was defined as findings from a single high quality randomised controlled trial. Conflicting evidence was defined as inconsistent findings among multiple trials.^39^ Study heterogeneity was assessed, based on methodological comparability, and disagreement resolved via discussion by the authors. Mean differences from individual studies were calculated via RevMan 5^38^ and presented to illustrate the individual study's populations’ data for symptom progression over time.

We attempted to contact corresponding authors where data was missing, or if it was not presented in a usable format. If no response was forthcoming, where possible we estimated the required parameters from other reported data, such as the standard deviation from the range.^41^

Effect size thresholds were taken from a recent analysis from tendinopathy literature which showed that: for pain effect, 0.45–0.86 was considered a small effect, 0.87–1.31 a medium effect, 1.32 or above a large effect. For function, 0.45–0.87 was considered a small effect, 0.88–1.52 a medium effect and 1.53 or above a large effect.^42^ For secondary proof of efficacy −0.07–0.23 was considered a small effect, 0.24–0.53 a medium effect and 0.54 and above a large effect.^43^ Effect sizes which did not reach the minimal small effect size threshold were reported as negligible.

## Results

### Search results

A total of 2825 studies were identified through electronic databases including grey literature (Figure 1). After duplicates were removed (*n* = 674), the remaining 2151 studies were screened. Based on title and abstract 2109 studies were excluded, with the full texts retrieved for the remaining 42 studies. Fifteen studies were excluded and the remaining 27 quality assessed with the PEDro scale and then the Cochrane Risk of Bias Tool 2.0.^44^ Three studies were excluded based on low methodological quality (<4/10) and 13 studies were excluded based on high risk of bias, therefore retaining 11 studies in the review.

**Figure 1.**
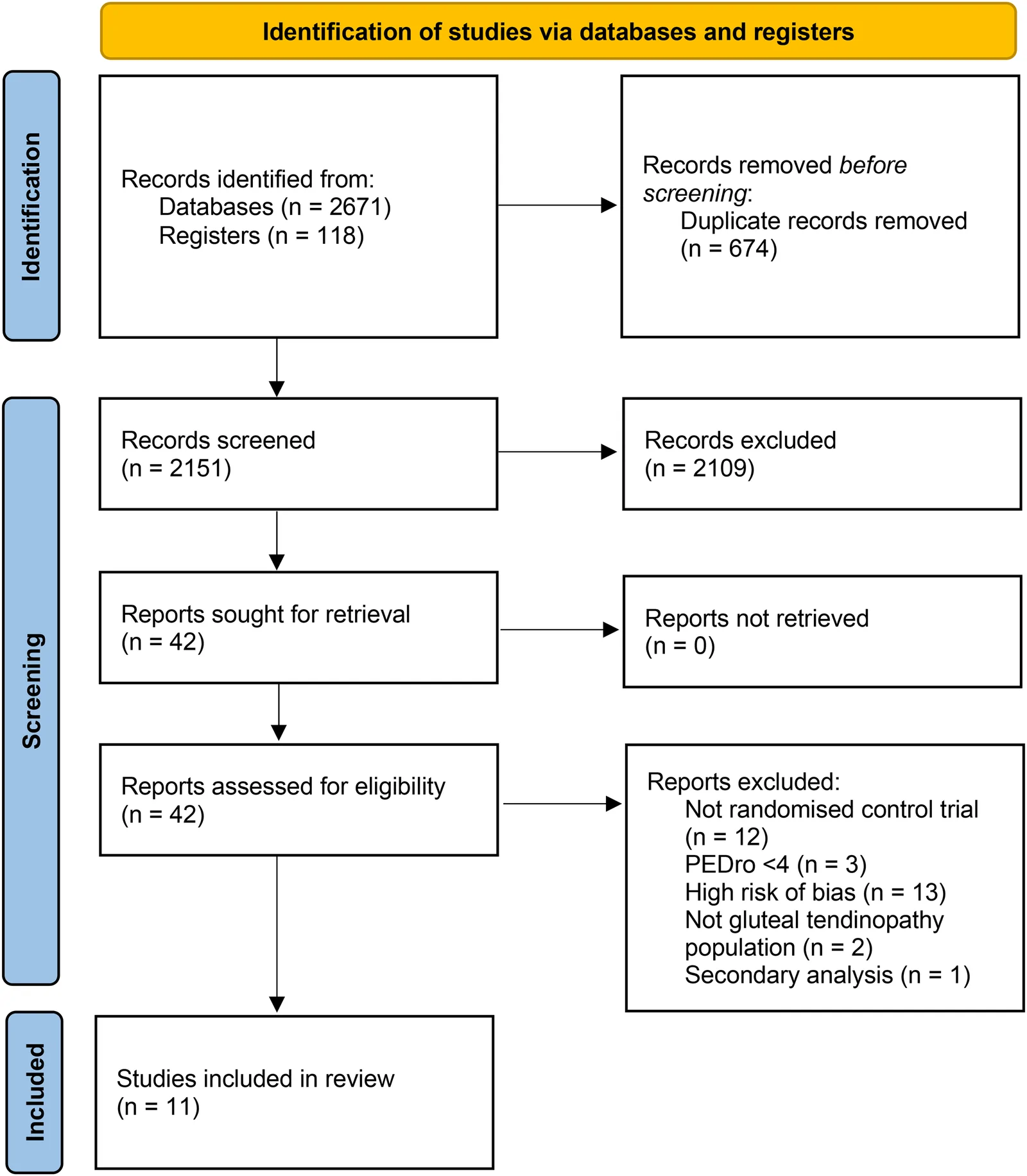
Identification and selection of studies.

### Methodological quality and risk of bias assessment

Of the 27 studies retrieved for this review, PEDro scores ranged from 3/10 to 10/10 (Table 1). Few studies achieved blinding of participants and assessors or clinicians,^24,45–49^ with only Fitzpatrick^22^ achieving blinding of all three groups. Almost half of the studies assessed failed to meet the allocation concealment and intention-to-treat criteria. Thirteen studies were considered to have a high risk of bias, four had some concerns, and the remaining seven were considered to have a low risk of bias (Table 2). The two criteria that often separated those of high risk of bias from those of low were deviations from intended intervention and measurement of the outcome. The three studies^50–52^ which scored <4 on the PEDro scale and the thirteen studies^21,24,45,49,53–61^ which scored high on risk of bias, were at this stage removed and played no further part in the analysis.

**Table 1.**
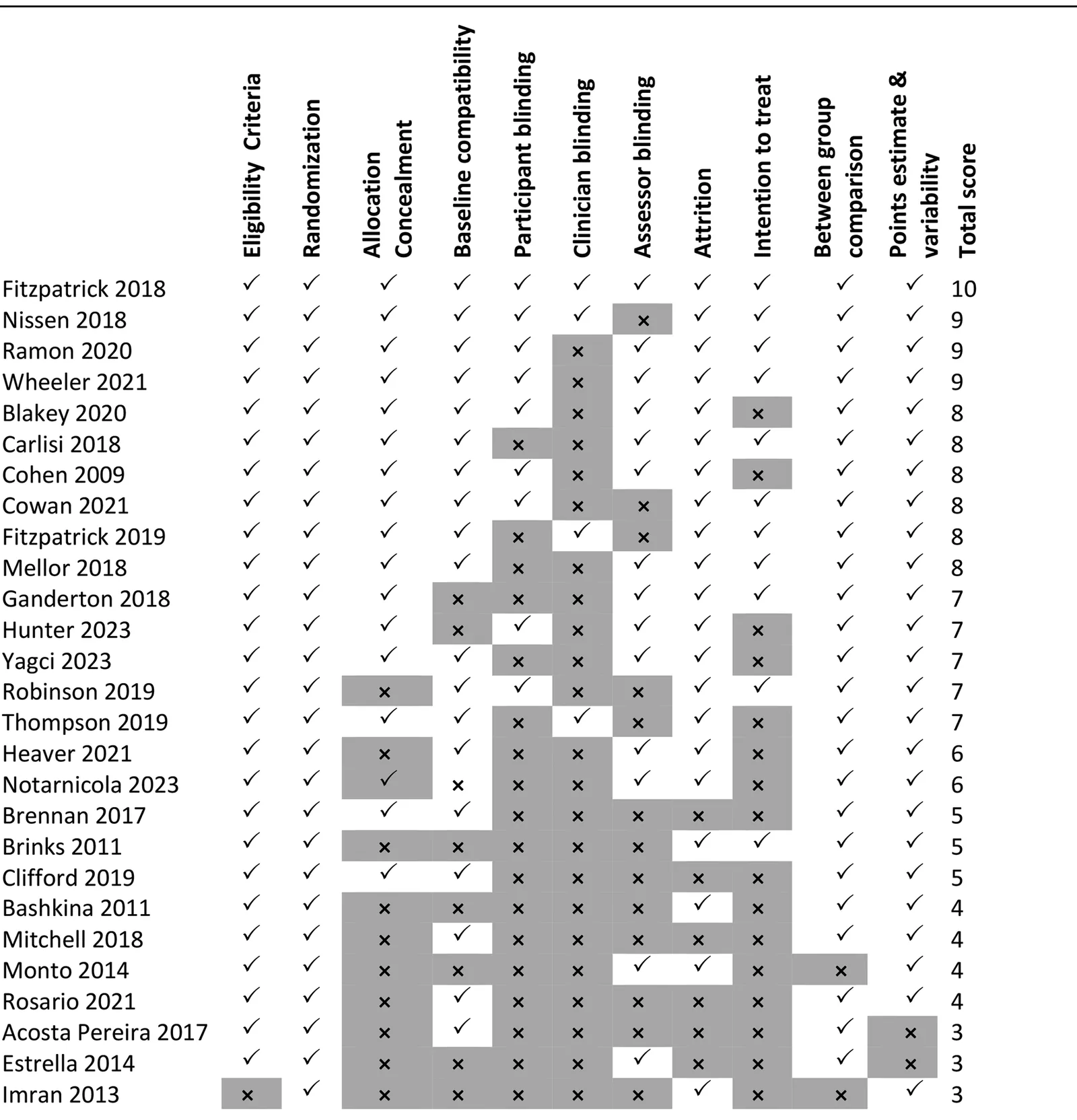
Quality assessment – PEDro scores.

**Table 2.**
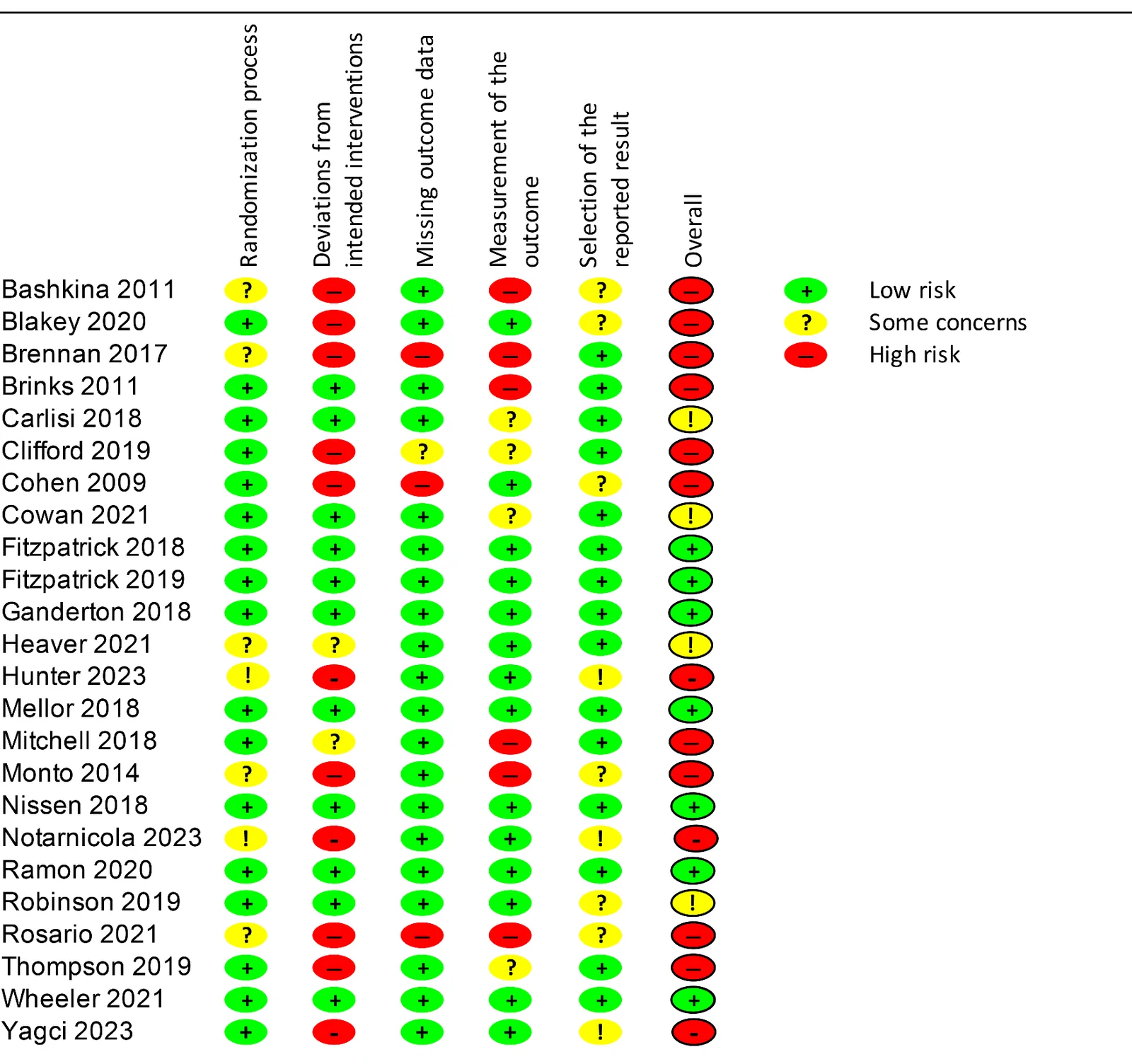
Risk of bias assessment.

### Study characteristics

Study characteristics including interventions, participant characteristics, outcome measures used, and key results, are presented in Table 3.

**Table 3.** Study characteristics.

Study	Interventions	Sample size; mean age (standard deviation); male, female	Outcome measures	Within group outcomes; mean score, (standard deviation), unless otherwise stated	Between group effects
**Carlisi (2018)**	Focused shockwave therapy 3 × 1 weekly Ultrasound daily for 10 days	n = 26; 61 (9.18); 5 M, 21F n = 24; 61.5 (9.52); 2 M, 22F	Numerical Pain Rating Scale Lower Extremity Functional Scale	Focused shockwave therapy scores improved from 5.12 (2.17) to 0.79 (1.28) at 6/12. Ultrasound scores improved from 4.93 (1.72) to 2.03 (2.09) at 6/12. Lower Extremity Functional Scale scores improved from 49 (19.7) to 68.21 (11.49) at 6/12. Ultrasound scores improved from 47.17 (17.94) to 63.39 (13.03) at 6/12.	No significant difference between groups at 6/12.
**Cowan** **(2021)**	Exercises/menopausal hormone treatment/education: Exercises/sham cream, education: Sham exercise/menopausal hormone treatment/education: Sham exercise/sham cream/education: 3/12 exercise programme 4 contacts with physiotherapist	*n* = 32; 58.31 (6.00) *n* = 31; 61.65 (7.50) *n* = 34, 62.00 (6.09) *n* = 35, 62.51 (5.80) 0 M 128F	The Victorian Institute of Sports Assessment Groin The hip disability and osteoarthritis outcome score pain subscale	Menopausal hormone treatment scores improved from 59.44 to 76.25 at 12/12. Placebo scores improved from 60.78 to 72.47 at 12/12. Globe exercises score improved from 60.77 to 76.51 at 12/12 Sham exercises scores improved from 59.45 to 72.20 at 12/12. Menopausal hormone treatment scores improved from 64.17 to 75.66 at 12/12. Placebo scores improved from 62.96 to 74.16 at 12/12. Globe scores improved from 63.20 to 76.05 at 12/12. Sham scores improved from 63.92 to 73.77 at 12/12.	No significant interaction effect between menopausal hormone treatment and exercise at 12/12
**Fitzpatrick (2018)**	Corticosteroid injection (blinded) Platelet-rich plasma injection (ultrasound guided) 1 × injection and exercise programme	*n* = 20; 59.7 (13.75); 1 M,19F *n* = 20; 60.3 (13.25) 3 M,17F	Modified Hip Harris Score	Corticosteroid injection scores improved from 54.15 (10.88) to 67.13 (16.04) at 3/12. Platelet-rich plasma injection scores improved from 53.77 (12.88) to 74.05 (13.92) at 3/12.	Significant improvement of platelet-rich plasma injection group at 3/12.
**Fitzpatrick (2019)**	Corticosteroid injection (ultrasound guided) Platelet-rich plasma injection (Ultrasound guided) 1× injection and exercise programme	*n* = 20: 59.7 (13.75);2 M,38 F *n* = 20: 60.3 (13.25); 6 M,34 F	Modified Hip Harris Score	Corticosteroid injection group scores improved from 54.15 (10.88) to 71.27 (25.78) at 104 weeks. Platelet-rich plasma injection group scores improved from 53.77 (12.88) 82.59 (9.71) at 104 weeks.	Significant improvement of platelet-rich plasma injection at 104 weeks.
**Ganderton (2018)**	GLoBE exercises and education Sham exercises and education 3/12 exercise programme	*n* = 46; 61.14 (6.70) 0 M, 46F *n* = 48; 62.53 (8.92) 0 M, 48F	The hip disability and osteoarthritis outcome score pain subscale The Victorian Institute of Sports Assessment Groin	GLoBE scores improved from 61.8 (15.3) to 75.6 (17.0) at 12/12. Sham exercise scores improved from 58.5 (15.7) to 74.4 (18.1) at 12/12. GLoBE scores improved from 61.60 (10.91) to 76.0 (17.4) at 12/12. Sham exercise scores improved from 53.83 (17.33) to 70.5 (21.6) at 12/12.	No significant difference found between groups at 12/12.
**Heaver** **(2021)**	Focused shockwave therapy 3 × 1 weekly corticosteroid injection (ultrasound guided) 1 × injection, supervised 12/12 exercise programme for both groups.	*n* = 48; 63.73 (11.87), 6 M, 42F *n* = 47; 60.31 (12.74), 4 M, 43F	Visual Analogue Scale Hip Harris Score	Focused shockwave therapy scores improved from 69.8 (16.9) to 37.1 (3.26) at 12/12. Corticosteroid injection scores improved from 70.1 (16.5) to 55.0 (2.87) at 12/12. Focused shockwave therapy scores improved from 52.03 (17.51) to 69.7 (22.0) at 12/12. Corticosteroid injection scores improved from 50.27 (14.22) to 57.5 (20.2) at 12/12.	Significant improvement at 12/12 for the focused shockwave therapy group at 12/12.
**Mellor (2018)**	Exercise combined with education 14 sessions. Corticosteroid injection 1 × injection Wait and see – nil intervention.	*n* = 69; 54.8 (8.1), 13 M, 56F *n = *66*;* 55.3 (9.4), 9 M, 57F *n* = 69; 54.5 (9.1), 15 M, 54F	Numerical Pain Rating Scale VISA G	Exercise combined with education scores improved from 4.8 (1.0) to 2.1 (2.2) at 12/12. Corticosteroid injection scores improved from 4.8 (1.0) to 2.3 (1.9) at 12/12. Wait and see scores improved from 4.9 (1.2) to 3.2 (2.6) at 12/12. Exercise combined with education scores improved from 60.2 (13.1) to 77.9 (16.5) at 12/12. Corticosteroid injection scores improved from 59.3 (11.1) to 72.8 (15.6) at 12/12. Wait and see scores improved from 60.2 (13.4) to 70.8 (18.0) at 12/12.	No significant difference in pain reduction or function improvement between exercise combined with education and corticosteroid injection groups at 12/12. Wait and see group did not improve at the same rate as exercise combined with education or corticosteroid injection.
**Nissen (2018)**	Corticosteroid injection SS 1 × injection	*N* = 21; 56.6 (14.6) 4 M, 17F *N* = 25; 59.6 (13.1), 3 M, 22F	NRPS	Corticosteroid injection pain scores reduced from 6.1 (1.5) by −3.0 (3.5) at 6/12. SS pain scores reduced from 6.6. (1.8) by −3.6 (3.1) at 6/12.	No significant difference between groups at 6/12.
**Ramon** **(2020)**	Focussed shock wave plus exercise programme. Sham focused shockwave therapy plus exercise programme: 3 × weekly focused shockwave therapy or sham focused shockwave therapy sessions, 6/12 exercise programme	*N* = 53; 57.1 (12.9), 11 M, 42F *N* = 50; 55.6 (11), 18 M, 32F	Visual Analogue Scale Hip Harris Score	Focussed shock wave scores improved from 6.3 (1.8) to 2.0 (2.1) at 2/12. Sham focused shockwave therapy scores improved from 6.3 (1.4) to 4.7 (2.1) at 2/12. Focused shock wave group improved from 65.0 (13.5) to 91.0 (10.3) at 6/12. Sham focused shockwave therapy scores improved from 65.9 (11.2) to 79.4 (12.5) at 6/12.	Significant improvement for focused shockwave therapy compared to sham group at 2/12.
**Robinson (2019)**	Active dynamic tape application Sham dynamic tape application. 1 × one-hour session	*N* = 50; 57.4 (13.8), 0 M, 50F Participants acted as their own control.	Numerical Pain Rating Scale	Active dynamic tape scores improved by mean (range) −1.9 (2, −5), from mean (SD) 4.5 (2.0) at 1/7. Sham dynamic tape scores improved by mean (range) −1.4 (2.5, −6), from mean (SD) 4.5 (2.0) at 1/7.	Active dynamic tape group demonstrated a statistically greater reduction in pain compared to sham group.
**Wheeler** **(2021)**	Radial extracorporeal shockwave therapy (recommended dose) Radial extracorporeal shockwave therapy (minimal dose), exercise programme. 3 × weekly ESWT, 6/12 exercise programme	*N* = 57; 61.7 (12.3), 8 M,49F *N* = 63; 60.0 (10.8), 15 M, 48F	Numerical Pain Rating Scale VISA G	Radial extracorporeal shockwave therapy (recommended dose) scores improved from 6.7 (1.5) to 4.6 (2.8) at 6/12. Radial extracorporeal shockwave therapy (minimal dose) scores improved from 6.1 (1.8) at to 4.0 (2.7) at 6/12. Radial extracorporeal shockwave therapy (recommended dose) scores improved from 45% ± 18% at baseline to 55% ± 22% at 6/12. Radial extracorporeal shockwave therapy (minimal dose) scores improved from 50% ± 16% to 62% ± 20% at 6/12.	No difference between groups at 6/12.

Study duration/data points x/12 = x months, x/52 = x weeks, x/7 = x/days.

Nine hundred and thirty-four participants were studied, of which 94.7% were female while three studies^23,62,63^ only included females. No study samples had an average participant age of less than 45 years old (range and mean SD), More than half the included studies (6/11, 54.5%) required participants to have had pain for three or more months, the two exceptions were Carlisi,^64^ greater than six weeks and Nissen,^46^ greater than one month. Three studies^23,62,63^ did not specify a length of time. Diagnosis was confirmed with clinical examination, plus imaging in six studies (54%),^22,47,48,64–36^ clinical examination only in five studies (46%).^23,37,45,46,63^

Eleven of 11 (100%) included studies reported separate measures of pain and function. Four^22,48,63,65^ (36%) reported combined measures of pain and function as their primary outcome measure. Visual Analogue Scale and Numerical Pain Rating Scale were the most used outcome measure for pain (100% of studies). Other outcomes of pain included the Lateral Hip Pain Questionnaire, Oxford hip score, Nonarthritic hip score and painDETECT. Outcomes of function included the Victorian Institute of Sports Assessment Groin, Patient Acceptable Symptom State, Hip Harris score, Patient Specific Functional Scale, Lower Extremity Functional Scale, Oswestry Low Back Disability Questionnaire, Lequesne, and Hip Dysfunction and Osteoarthritis Outcome Score (Table 3).

## Evidence of efficacy

A summary of findings concerning efficacy and strength of evidence is found in Table 4.

**Table 4.** Efficacy and strength of evidence for interventions considered for primary and secondary proof of efficacy in the form of an ‘evidence map’.

Intervention	Outcome domain	Short term (0–3 months)	Medium term (3–6 months)	Long term (6–12 months)
Primary proof of efficacy				
Corticosteroid injection	Pain effects SMD [95% CI]	0.51 [0.16, 0.86] Mellor (2018)* n = 129 *Moderate strength evidence* *Small effect size*	0.22 [−0.14, 0.57] Mellor (2018)* n = 124 *Moderate strength evidence* *No effect*	0.39 [0.04, 0.75] Mellor (2018)* n = 124 *Moderate strength evidence* *Negligible effect*
	Pain outcomes MD [95% CI]	2.30 [1.74,2.86] Mellor (2018)*		2.50 [1.97, 3.03] Mellor (2018)*
	Function: effects: SMD [95% CI]	0.36 [0.01, 0.71] Mellor (2018)* n = 129 *Moderate strength evidence* *Negligible effect*	0.28 [−0.07, 0.63] Mellor (2018)* n = 125 *Moderate strength evidence* *No effect*	0.12 [−0.23, 0.47] Mellor (2018)* n = 124 *Moderate strength evidence* *No effect*
	Function outcomes: MD [95% CI]	11.40 [6.80, 16.00] Mellor (2018)*		
Exercise combined with education	Pain effects: SMD [95% CI]	0.95 [0.58, 1.33] Mellor (2018)* n = 122 *Moderate strength evidence* *Medium effect size*	0.60 [0.23, 0.96] Mellor (2018)* *n* = 122 *Moderate strength evidence* *Small effect size*	0.46 [0.10, 0.81] Mellor (2018)* n = 126 *Moderate strength evidence* *Small effect size*
	Pain outcomes: MD [95% CI]	3.10 [2.60,3.60] Mellor (2018)* 13.00 [5.80, 20.20] Ganderton (2018)*	3.00 [2.47, 3.53] Mellor (2018)*	2.70 [2.12, 3.28] Mellor (2018)* 13.80 [6.72, 20.88] Ganderton (2018)*
	Function effects: SMD [95% CI]	0.91 [0.53, 1.28] *Mellor (2018*)* *n* = 122 *Moderate strength evidence* *Medium effect size*	0.79 [0.42, 1.16] Mellor (2018)* n = 122 *Moderate strength evidence* *Small effect size*	0.41 [0.05, 0.76] Mellor (2018)* *n* = 126 *Moderate strength evidence* *Negligible effect*
	Function outcomes: MD [95% CI]	19.10 [14.29, 23.91] Mellor (2018)* 11.50 [5.11, 17.89] Ganderton (2018)*	19.00 [14.09, 23.91] Mellor (2018)*	17.70 [12.62, 22.78] Mellor (2018)* 14.40 [7.98, 20.82] Ganderton (2018)*
Secondary proof of efficacy				
Focused shockwave therapy plus exercise combined with education compared to corticosteroid injection plus exercise combined with education	Pain effects: SMD [95% CI]	0.16 [−0.24, 0.56] Heaver (2021)* n = 98 *Moderate strength evidence* *No difference in effect*		5.77 [4.84, 6.71] Heaver (2021)* n = 94 *Moderate strength evidence* *Large effect size* *focused shockwave therapy superiority in effect*
	Pain: outcomes: MD [95% CI]	3.04 [1.86, 4.22] Carlisi (2018)* 25.80 [17.36, 34.24] Heaver (2021)* 4.30 [3.55, 5.05] Ramon (2020)*		32.70 [28.06, 37.34] Heaver (2021)*
	Function effects: SMD [95% CI]	0.23 [−0.17, 0.62] Heaver (2021)* n = 104 *Moderate strength evidence* *No difference in effect*		
	Function outcomes: MD [95% CI]	16.08 [7.33, 24.83] Carlisi (2018)* 15.37 [8.33, 22.41] Heaver (2021)* 25.40 [20.81, 29.99] Ramon (2020)*		
Platelet-rich plasma injection plus exercise combined with education compared to corticosteroid injection plus exercise combined with education	Pain effects: SMD [95% CI]			
	Pain outcomes: MD [95% CI]			
	Function effects: SMD [95% CI]	0.46 [0.00, 0.91] Fitzpatrick (2018)* n = 76 Moderate strength evidence Medium effect size Superiority in effect		
	Function outcomes: MD [95% CI]	20.28 [14.53, 26.03] Fitzpatrick (2019)*		

*Note:* SMD: standardised mean difference; MD: mean difference; 95% CI: 95% confidence interval.

Effect size for pain effects primary proof: small = 0.45–0.86, medium = 0.87–1.31, large = 1.32 or above.

Effect size for function effects primary proof: small = 0.45–0.87, medium = 0.88–1.52, large = 1.53 or above.

Effect size for pain and function secondary proof: small = −0.07–0.23, medium = 0.24–0.53, large = 0.54 or above.

Strong evidence is represented by dark green, moderate: lighter green, no data: grey.

* denotes (*in italics)* authors of source paper used for comparison.

### Primary proof of efficacy

#### Corticosteroid Injection

All data for primary proof of efficacy for pain and function for corticosteroid was derived from the Mellor et al.^36^ data only and is reported as SMD. Primary proof of efficacy is based on a comparison between the corticosteroid treatment arm and a wait-and-see group within the same study.^36^

For pain, there was moderate strength evidence that corticosteroid injection^36^ has a small effect on pain in the short-term (SMD 0.51, 95% CI [0.16, 0.86]),^36^ moderate strength evidence of no effect on pain in the medium-term (SMD 0.22, 95% CI [−0.14, 0.57]),^36^ and moderate strength evidence of negligible effect on pain in the long-term (SMD 0.39, 95% CI [0.04, 0.75])^36^ (Table 4).

For function, there was moderate strength evidence that corticosteroid injection^36^ has a negligible effect on function in the short-term (SMD 0.36, 95% CI [0.01, 0.71]),^36^ with moderate strength evidence of no effect in the medium-term (SMD 0.28, 95% CI [−0.07, 0.63]),^36^ and long-term (SMD 0.12, 95% CI [−0.23, 0.47])^36^ (Table 4).

Five other studies^15,22,45,46,65^ also evaluated the efficacy of corticosteroid injection. Methodological quality and risk of bias varied across the six studies (PEDro range 6–10/10; risk of bias range low to some concern).

#### Exercise and education

All data for primary proof of efficacy for pain and function for exercise and education was derived from the Mellor et al.^36^ data only and is reported as SMD. This analysis compared the exercise and education treatment arm with a wait-and-see group within the same study.^36^

For pain, there was moderate strength evidence that exercise and education^36^ has a medium effect in the short-term (SMD 0.95, 95% CI [0.58, 1.33]),^36^ moderate strength of small effect in the medium-term (SMD 0.60, 95% CI [0.23, 0.96]),^36^ and moderate strength evidence of small effect in the long-term (SMD 0.46, 95% CI [0.10, 0.81])^36^ (Table 4).

For function, there was moderate strength evidence that exercise and education^36^ has a medium effect in the short-term (SMD 0.91, 95% CI [0.53, 1.28]),^36^ moderate strength evidence of small effect in the medium-term (SMD 0.79, 95% CI [0.42, 1.16]),^36^ and moderate strength evidence of negligible effect in the long-term (SMD 0.41, 95% CI [0.05, 0.76])^36^ (Table 4).

Two further studies^23,63^ evaluated the efficacy of exercise therapy, as a primary intervention, one^23^ in combination with education, and another^63^ in combination with hormone replacement therapy and education. Methodological quality and risk of bias varied across the three studies (PEDro range 7–8/10; risk of bias range low to some concern).

Secondary proof of efficacy determined by comparison to interventions with primary proof of efficacy.

#### Shockwave therapy

Four studies^37,47,48,64^ evaluated the efficacy of shockwave therapy as a primary intervention. Methodological quality was high across the four studies (PEDro range 6–9/10; risk of bias range some concern – low risk).

Secondary proof of efficacy for pain and function for shockwave therapy was derived using data from Heaver et al.^37^ only and reported as the SMD. This analysis compared the treatment arms of focused shockwave therapy combined with an exercise programme versus corticosteroid injection combined with an exercise programme within the same study.^37^

There was moderate strength evidence of no difference in effect of focused shockwave therapy on pain in the short-term compared to corticosteroid injection (SMD 0.16, 95% CI [−0.24, 0.56]),^37^ and moderate strength evidence with a large effect size (SMD 5.77, 95% CI [4.84, 6.71])^37^ of superiority of focused shockwave therapy in the long-term (Table 4). There was moderate strength evidence of no difference in effect of focused shockwave therapy on function in the short term compared to corticosteroid injection (SMD 0.23, 95% CI [−0.17, 0.62]).^37^

#### Platelet-rich plasma

Two studies^22,65^ from one project evaluated the efficacy of platelet-rich plasma injection compared to corticosteroid injection, with an associated exercise programme, as a primary intervention. Methodological quality was high for both studies (PEDro range 8–10/10; risk of bias low risk). Both studies^22,65^ used the same patient demographic, double reporting the data.

Secondary proof of efficacy for pain and function for platelet-rich plasma injections was derived using data from the first published results from Fitzpatrick et al.^22^ only and reported as the standard mean difference. This analysis compared the treatment arms of platelet-rich plasma injection with an exercise programme versus corticosteroid injection combined with an exercise programme within the same study.^22^

For function, there was moderate strength evidence with a small effect size (SMD 0.46, 95% CI [0.00, 0.91])^22^ of superiority in effect of platelet-rich plasma injection^22^ compared to corticosteroid injection^22^ in the short term. Long-term comparisons were not possible due to the cross over study design used in the trial.^65^

## Discussion

A key finding of this systematic review is that it establishes moderate strength evidence proof of efficacy that a continuous, progressive, supervised targeted exercise programme in conjunction with periodic, timely education and checking of learning by a health professional has a positive effect at all time points.^23,36,55^ Previous research has demonstrated that exercise has a beneficial effect on tendinopathies.^66^ Exercise has a positive effect on the biochemical make up of tendons and tissue repair^67^ and appears to have an analgesic short-term effect,^68^ reduces kinesiphobia,^69^ and has a positive impact on psychosocial issues arising from chronic pain.^70^ Patient education needs to contain several key elements to be successful.^23,36^ This included specific information around behaviour change, such as avoiding compressive and tensile forces of the medial gluteal tendon and adequate load management for daily activities, education concerning the condition and its management, in conjunction with appropriate exercises in the form of a responsive personalised exercise regime.^71,72^ All treating physiotherapists in the successful trials within this systematic review^23,36^ had been specifically trained to provide education and exercise, with all sessions delivered face-to-face and augmented with easy to access take home information, supported by various media.^23,36^

The moderate strength evidence findings of corticosteroid injection in this review echo previous systematic review findings,^16,19^ that corticosteroid injection demonstrates a pain reducing effect in the short term, with this effect diminishing in the longer term. Early tendinopathies display some signs of the classic inflammatory process,^73^ whereas chronic tendinopathies display hypoxic areas and abnormal histology.^74^ This may go some way to explaining why patients with inflammation processes, associated bursitis or early stages of gluteal tendinopathy respond to corticosteroid injection and this effect lessens as chronicity of the tendon pathology ensues.

This systematic review was able to determine moderate strength evidence of proof of efficacy for focused shockwave therapy in combination with a concurrent exercise programme.^37,47^ This makes focused shockwave therapy a viable alternative for non-invasive, inexpensive pain relief and improved function. These findings mirror those found for other tendon pathologies.^75^ Patient-reported outcomes^71^ in a demographic of chronic gluteal tendinopathy patients are largely favourable of the use of shockwave for pain. Moderate strength evidence of a small effect in the short term for platelet-rich plasma injection was demonstrated.^22^ However, it is difficult to make best practice clinical management suggestions based on a single study with large confidence intervals and short-term results only.

The synthesised evidence presented in this systematic review is of moderate strength and needs to be interpreted with this limitation in mind. The use of heterogeneous interventions within the individual studies did not allow for pooling of the results for meta-analysis leading to a reduced ability to conclude categorically from their results.^76^

The trials included in this systematic review used varying eligibility criteria for inclusion and therefore there is limited certainty that the populations included were consistently and accurately diagnosed.^77,78^ The study populations may also not truly reflect the demographic of patients presenting in daily clinic thereby limiting generalisability. The wide confidence intervals reported for the individual modalities also adds inherent uncertainty to the precision of their efficacy^34^ and to the utility of the modality, therefore caution is warranted when considering the results of this systematic review. The Cochrane Risk of Bias 2 assessment tool has been shown to have poor inter-rater reliability^79,80^ and although care was taken to adhere to The Cochrane Risk of Bias 2 scoring guidance provided by the Cochrane centre, this limitation must be considered when interpreting the results. Finally, we have interpreted effect sizes according to new data derived from analysis of the existing tendinopathy literature, which is preferable to using Cohen's criteria, however a further improvement would be for that interpretation to be anchored on clinically validated effect sizes.

**Clinical message**

- A combined approach of graded exercises and education has the best evidence for the management of gluteal tendinopathy and should be adopted as the core approach.
- Corticosteroid injection alongside exercise assists with pain control in the short term.
- Focussed shock wave may add additional benefit for pain control over the longer term when compared to corticosteroid.
- Platelet-rich plasma injections demonstrate a superior effect for function when compared to corticosteroid.

## Supplemental Material

sj-docx-1-cre-10.1177_02692155251327298 - Supplemental material for The efficacy of gluteal tendinopathy treatments: A systematic review Supplemental material, sj-docx-1-cre-10.1177_02692155251327298 for The efficacy of gluteal tendinopathy treatments: A systematic review by Tobias Bremer, Peter Nicklen, Angie Fearon and Dylan Morrissey in Clinical Rehabilitation

sj-docx-2-cre-10.1177_02692155251327298 - Supplemental material for The efficacy of gluteal tendinopathy treatments: A systematic review Supplemental material, sj-docx-2-cre-10.1177_02692155251327298 for The efficacy of gluteal tendinopathy treatments: A systematic review by Tobias Bremer, Peter Nicklen, Angie Fearon and Dylan Morrissey in Clinical Rehabilitation

## Data Availability

The datasets generated during and/or analysed during the current study are available from the corresponding author on reasonable request.
