# Supplementary material for: The efficacy of gluteal tendinopathy treatments: A systematic review
Source: Clin Rehabil. 2025 Apr 13;39(5):600–17. doi: 10.1177/02692155251327298 (PMC13687622; doi:10.1177/02692155251327298)
Supplement: Supplementary file 1 [file sj-docx-1-cre-10.1177_02692155251327298.docx]

**TERMS FOR PATHOLOGY: GTPS**

Greater trochanteric pain OR

Greater trochanter pain OR

Lateral Hip pain OR

Trochanteric pain OR

Lateral trochanteric pain OR

Greater trochanteric pain syndrome OR

Greater trochanter pain syndrome OR

GTPS OR

Greater trochanteric bursitis OR

Trochanteric bursitis OR

Trochanteric tendinitis OR

Trochanteric tendinosis OR

Trochanteric tend?nopathy OR

Gluteus medius tend?nopathy OR

Gluteus medius tendinitis OR

Gluteus medius tendinosis OR

Gluteal tend?nopathy OR

Gluteal tendinitis OR

Gluteal tendinosis OR

Gluteus minimus tend?nopathy OR

Gluteus minimus tendinitis OR

Gluteus minimus tendinosis OR

Gluteus maximus tend?nopathy OR

Gluteus maximus tendinitis OR

Gluteus maximus tendinosis OR

Hip abductor tend?nopathy OR

Hip abductor tendinitis OR

Hip abductor tendinosis OR

Hip tend?nopathy OR

Hip tendinitis OR

Hip tendinosis OR

Hip bursitis OR

Trochanteric bursa OR

Subgluteal bursa OR

Subgluteus-maximus OR

Subgluteus-medius OR

Subgluteus-minimus OR

Gluteal enthesopathy OR

Gluteus medius enthesopathy OR

Gluteus medius enthesopathy OR

Gluteus minimus enthesopathy OR

Trochanteric bursopathy OR

Subgluteal bursitis OR

Trochanteritis OR

Peritrochanteric bursitis

**TERMS FOR INTERVENTION/TREATMENT MODALITIES**

Physiotherapy OR

physical-therapy OR

exercise OR

training OR

rehabilitation OR

strength-training OR

Therapy OR

Sports therapy OR

Osteopathy OR

Chiropractic therapy OR

primary-care OR

treatment OR

conservative OR

non-operative OR

intervention OR

management OR

multiple-treatment OR

functional-outcome OR

usual care OR

pain-management OR

Twice? pain management OR

pain relief OR

multidisciplinary OR

occupational-therapy OR

adjustment OR

mechanotherapy OR

mobile?ation OR

immobili?ation OR

manual-therapy OR

range of motion OR

biomechanic OR

range-of-motion OR

acupuncture OR

massage OR

electrotherapy OR

cognitive-behavioural-therapy OR

Twice? biomechanic OR

advice OR

bracing OR

brace OR

taping OR

soft tissue therapy OR

Soft tissue release OR

soft-tissue-mobili?ation OR

STTM OR

soft-tissue OR

myofascial OR

proprioception OR

off-loading OR

loading OR

eccentric OR

eccentric loading OR

strengthening OR

balance OR

power OR

resistance OR

isometric OR

concentric OR

concentric loading OR

stretch* OR

theraband OR

rest OR

activity modifications OR

weight reduction OR

orthotics OR

emt OR

shockwave therapy OR

SWT OR

extracorporeal shockwave therapy OR

ESWT OR

therapeutic ultrasound OR

pens OR

nerve-block OR

nitric oxide OR

Nitric oxide donor therapy OR

topical glyceryl trinitrate OR

TGTN OR

patches OR

NSAIDs OR

non steroidal anti-inflammatory drugs OR

Steroid injections OR

inject* OR

laser therapy OR

platelet-rich-plasma OR

PRP OR

Stem cell OR

sclerotherapy OR

sclerosing OR

prolotherapy OR

proliferation therapy OR

operative OR

surgical OR

surgery OR

operation OR

surgical therapy OR

shaving OR

arthroscopy OR

release OR

bursectomy OR

repair OR

open OR

percutaneous

**TERMS FOR TYPES OF TRIALS**

Randomi?ed-control-trial OR

controlled OR

comparison OR

comparator OR

intervention OR

randomi?ed OR

placebo-controlled OR

Placebo OR

double-blind OR

clinical-study OR

efficacy OR

multiple-subjects

MESH TERMS: hip, bursitis, tendinopathy, enthesopathy
