# Supplementary material for: The efficacy of gluteal tendinopathy treatments: A systematic review
Source: Clin Rehabil. 2025 Apr 13;39(5):600–17. doi: 10.1177/02692155251327298 (PMC13687622; doi:10.1177/02692155251327298)
Supplement: Supplementary file 2 [file sj-docx-2-cre-10.1177_02692155251327298.docx]

Appendix 1: Studies excluded from meta analysis.

| Study | Interventions Compared | Population Characteristics | Outcome Measures | Pooling Possible |
| --- | --- | --- | --- | --- |
| Fitzpatrick (2018) | Platelet-Rich Plasma Injections vs. Corticosteroid Injections + exercise | **Corticosteroid Group (n = 40):** Age: 59.7 y (23–78), Male: 2 (5%), Female: 38 (95%), BMI: 26.96 ± 4.33 (18.8–39.5)  **PRP Group (n = 40):** Age: 60.3 y (23–76), Male: 6 (15%), Female: 34 (85%), BMI: 28.42 ± 4.58 (20.0–43.9) | Modified Hip Harris Score (mHHS), PASS | **No**: Both Fitzpatrick studies (2018&2019) use the same population. |
| Fitzpatrick (2019) | Platelet-Rich Plasma Injections vs. Corticosteroid Injections + exercise | Same as Fitzpatrick (2018) | Modified Hip Harris Score (mHHS), PASS | **No**: Both Fitzpatrick studies (2018&2019) use the same population. |
| Ganderton (2018) | 12-week exercise programs (GLoBE vs. sham exercise program) | **GLoBE (n = 46):** Age: 61.14 ± 6.70, Height: 164.40 ± 6.10 cm, Mass: 71.83 ± 12.80 kg, BMI: 26.57 ± 4.69 kg/m², VISA-G Score: 61.60 ± 10.91, Side affected (left/right): 23/20 **Sham (n = 48):** Age: 62.53 ± 8.92, Height: 163.09 ± 6.60 cm, Mass: 81.92 ± 20.04 kg, BMI: 30.83 ± 7.18 kg/m², VISA-G Score: 53.83 ± 17.33, Side affected: 25/22  **100% participants post-menopausal females for both groups** | VISA-G, HOOS (pain subscale) | **No**: After lengthy analysis of the exercise programs included within the individual studies by Mellor (2018) and Ganderton (2018), we have concluded that the individual studies differ significantly in approach and delivery and are not comparable.  Only post-menopausal females included in Ganderton (2018) study. Not comparable to participant characteristics from Mellor (2018) study. |
| Mellor (2018) | Exercise and education vs. Corticosteroid Injection vs. Wait-and-See approach | **All Participants (n = 204):** Age: 54.8 (8.8) years, Female: 167 (81.9%), Height: 1.7 (0.1) m, Weight: 75.9 (15.3) kg, BMI: 27.4 (5.1) **Education plus Exercise (n = 49):** Age: 54.8 (8.1) years, Female: 56 (81.2%), Height: 1.7 (0.1) m, Weight: 75.9 (14.4) kg, BMI: 27.7 (4.8) **Corticosteroid Injection (n = 66):** Age: 55.3 (9.4) years, Female: 57 (86.4%), Height: 1.7 (0.1) m, Weight: 74.4 (14.6) kg, BMI: 27.0 (5.1) **Wait-and-See (n = 69):** Age: 54.5 (9.1) years, Female: 54 (78.3%), Height: 1.7 (0.1) m, Weight: 77.3 (16.7) kg, BMI: 27.6 (6.5) | NRS, VISA-G | **No**: See above |
| Wheeler (2021) | Radial extracorporeal shockwave therapy (recommend dose) and exercise protocol vs. Radial extracorporeal shockwave therapy (minimal dose) and same exercise protocol.  Each group received 3x weekly r-ESWT, 6/12 exercise protocol. | **F-ESWT (n = 57):** Age: 61.7 ± 12.3 years, Female: 49 (86%), Symptom duration: 50.1 ± 62.9 weeks  **Control (n = 63):** Age: 60.0 ± 10.8 years, Female: 48 (76%), Symptom duration: 41.2 ± 43.3 weeks | NRS pain, SF-36 scores | No:   Unable to pool as the interventions compared are not comparable with any of the other included studies. |
| Ramon (2020) | Focused Extracorporeal Shock Wave Therapy (F-ESWT) and exercise protocol vs. sham F-ESWT and same exercise protocol | **F-ESWT (n = 53):** Age: 57.1 ± 12.9 years, Female: 42 (79%), Right side: 30 (57%)  **Control (n = 50):** Age: 55.6 ± 11 years, Female: 32 (64%), Right side: 28 (56%) | VAS, HHS, LEFS, EQ-5D | No:   Unable to pool as the interventions compared are not comparable with any of the other included studies. |
| Nissen (2018) | Glucocorticoid injections vs. Saline solution injection | **Active Treatment (n = 21):** Age: 56.6 (14.6) years, Female: 81%, Weight: 74.4 (15.1) kg, Height: 162 (159, 168) cm, BMI: 27.9 (6.1), Pain (24 h): 6.1 (1.5), Pain (palpation): 6.6 (2.0), Past Injection (% yes): 38.1%, SF-12: 35.3 (8.3), Oswestry: 39.9 (13.1), WOMAC pain: 251.4 (80.5), WOMAC function: 414.6 (154.9)  **Placebo Group (n = 25):** Age: 59.6 (13.1) years, Female: 88%, Weight: 74.7 (15.8) kg, Height: 160 (153, 164) cm, BMI: 28.8 (4.9), Pain (24 h): 6.6 (1.8), Pain (palpation): 7.1 (1.9), Past Injection (% yes): 40%, SF-12: 34.8 (8.3), Oswestry: 39.9 (14.9), WOMAC pain: 247.2 (87.2), WOMAC function: 366.1 (175.7) | SF-12, Oswestry, WOMAC pain/function | No:  Not reported well – baseline not clear and then only change scores reported. Requested raw data sets which were incomprehensible. |
